# Energy-Optimal Adaptive Control Based on Model Predictive Control

**DOI:** 10.3390/s23094568

**Published:** 2023-05-08

**Authors:** Yuxi Li, Gang Hao

**Affiliations:** 1 School of Electronic Engineering, Heilongjiang University, Harbin 150080, China; 2211763@s.hlju.edu.cn; 2 Key Laboratory of Information Fusion Estimation and Detection, Harbin 150080, China

**Keywords:** model predictive control (MPC), cubature Kalman filter, energy-optimal cruise control, artificial neural network

## Abstract

Energy-optimal adaptive cruise control (EACC) is becoming increasingly popular due to its ability to save energy. Considering the negative impacts of system noise on the EACC, an improved modified model predictive control (MPC) is proposed, which combines the Sage-Husaadaptive Kalman filter (SHAKF), the cubature Kalman filter (CKF), and the back-propagation neural network (BPNN). The proposed MPC improves safety and tracking performance while further reducing energy consumption. The final simulation results show that the proposed algorithm has a stronger energy-saving capability compared to previous studies and always maintains an appropriate relative distance and relative speed to the vehicle in front, verifying the effectiveness of the proposed algorithm.

## 1. Introduction

Electric and hybrid vehicles are gaining popularity as environmentally friendly alternatives to conventional fuel vehicles due to their reduced emissions and lower operating costs [1,2,3]. However, electric vehicles face issues such as limited range, extended charging times, and high battery costs [4]. As a result, it is crucial to develop eco-driving assistance systems that optimize energy consumption in electric vehicles while maintaining safety and comfort [5].

Adaptive cruise control (ACC) is a widely used driving assistance system that automatically adjusts a vehicle’s speed and maintains a safe distance from the vehicle ahead. Nevertheless, conventional ACC systems have limitations in terms of energy efficiency, driver comfort, and tracking stability. To address these challenges, researchers have proposed various ACC extensions, including cooperative adaptive cruise control (CACC) [6,7,8], personalized adaptive cruise control (PACC) [9,10,11], and energy-optimal adaptive cruise control (EACC) [1,12,13,14,15]. CACC enables multi-vehicle cooperation through vehicle communication systems, while PACC tailor sand simulates individual driving habits. EACC focuses on energy consumption and aims to conserve energy while tracking the leading vehicle. Several studies have applied model predictive control (MPC) to ACC problems, yielding promising results [12,13].

EACC is a crucial aspect of ACC, but it faces challenges due to the uncertainty of the leading vehicle’s state. One approach to this issue involves combining MPC and dynamic programming (DP) algorithms. Weißmann A et al. use DP to plan the speed and route from the cloud based on the starting position, while the host vehicle uses the cloud information to estimate the leading vehicle’s speed in real time and applies MPC for calculation and control [14]. Alternatively, Pan C et al. combine an economical linearized energy consumption equation with MPC, proposing the use of a nonlinear auto-regressive model with exogenous inputs (NARX) to predict the leading vehicle’s speed [15]. Similar methods employ the conditional linear Gauss (CLG) model to predict the leading vehicle’s speed and control the host vehicle using chance constraints, stochastic MPC, and randomized MPC [7].

Some existing ACC studies only consider vehicle measurement noise [16], while most others disregard noise altogether [1,7,12,13,14]. A few papers assume the use of filters for preprocessing [17]. ACC algorithms for cars with ultrasonic sensors have been researched and considered [18], but these sensors have limited range and accuracy and can only measure vehicle distance. Noise is pervasive and can significantly impact ACC performance. Inaccurate system process state estimation can lead to control system instability, resulting in frequent acceleration and deceleration and wasting energy. Hence, it is essential to develop EACC algorithms capable of handling noisy conditions.

Some recent studies have considered control systems with noise. Aubeck F et al. addressed the plug-in hybrid vehicle energy management problem using a generalized stochastic particle filtering algorithm for filtering, followed by a two-level MPC for coordinated vehicle fuel use and charging management [19]. Another example is [20], where Yan D et al. combined a Kalman-consistent filter and a fixed-time disturbance observer with a multi-constraint MPC strategy to control the formation flight of unmanned aerial vehicles. To solve the problem above, an EACC system algorithm is proposed for solving vehicle systems with noise. The main contributions of this paper are as follows:

(1) In order to address the effects of process noise and measurement noise in vehicle nonlinear systems, a Sage-Husa adaptive cubature Kalman filter (SHACKF) is proposed. By filtering, the leading vehicle’s speed prediction model is improved to tackle the issue of diminished multi-step prediction accuracy.
(2) Secondly, a back-propagation neural network (BPNN) for trend prediction is incorporated, which can be combined with various leading vehicle speed prediction models. Additionally, the energy consumption equation and kinetic energy recovery system (KERS) are considered in order to address the problem of frequent deceleration due to noise. The control is limited to a range that excludes mechanical braking, and interpolation is used to fit the motor’s efficiency. This is combined with MPC to calculate the most energy-efficient operating point.

Comparison experiments were conducted under two types of cycles, and the results show that the proposed algorithm significantly improves energy savings, following distance, and following relative speed compared to previous studies.

The rest of this paper is organized as follows: the system model is presented in Section 2; the filter and controller are designed in Section 3; the simulation results are shown in Section 4; and the conclusions are given in Section 5.

## 2. Modeling

In this paper, we focus on a vehicle-following scenario on a single lane, aiming to control the host vehicle to maintain an optimal distance from the front vehicle while minimizing energy consumption. To implement the algorithm, the first step is to model the vehicle cruise process equation, measurement equation, and energy consumption equation.

### 2.1. Vehicle Longitudinal Dynamics Model

**Figure 1:**
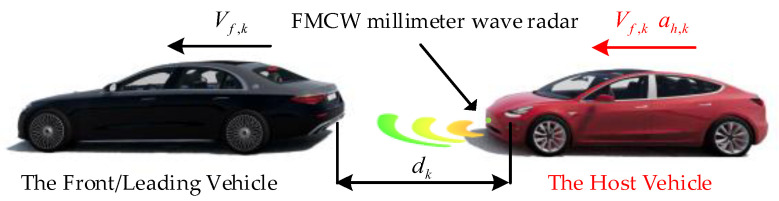
Schematic of two consecutive vehicles.

Figure 1 depicts a schematic illustration of two consecutive vehicles in the traffic flow, where k represents discrete time, $V_{f,k}$ represents the velocity of the front vehicle, $d_{k}$ represents the distance difference between the two vehicles, $V_{h,k}$ denotes the velocity of the host vehicle, and $a_{h,k}$ denotes the acceleration of the host vehicle. The considered system process vector $x_{k}^{}$ can be modeled as follows:

(1) $$x_{k}^{}={\left[\begin{array}{cccc} V_{f,k} & d_{k} & V_{h,k} & a_{h,k} \end{array}\right]}^{T}$$

Firstly, the state of the host vehicle is considered. Numerous papers have proposed models for the vehicle-following scenario [1,21]. Specifically, the relationship between the velocity and control input of the host vehicle is as follows:

(2) $$ma_{h,k}=\frac{T_{m}\left(u_{k}\right)i\eta }{r}-F_{rolling}-F_{air}\left(V_{h,k}\right)-F_{grade}$$

(3) $$V_{h,k+1}=V_{h,k}+T_{s}a_{h,k}$$

where m denotes the vehicle mass, $T_{s}$ is the sampling and control period, $T_{m}\left(u_{k}\right)$ is the motor output torque affected by control input $u_{k}$, which will be described in detail in Section 2.3, i is the transmission ratio product of the gearbox and the main reducer, η is the transmission system efficiency, and r is the wheel radius. The resistances $F_{rolling}$, $F_{air}\left(V_{h,k}^{}\right)$, and $F_{grade}$ represent the rolling resistance, the air resistance function, and the grade resistance, respectively [1,21]. They are calculated as follows:

(4) $$F_{rolling}=mgf_{r}\cos\alpha$$

(5) $$F_{air}\left(V_{h,k}^{}\right)=\frac{C_{w}\rho A_{window}V_{h,k}^{2}}{2}$$

(6) $$F_{grade}=mg\sin\alpha$$

where $f_{r}$ is the rolling resistance coefficient, g is the gravitational acceleration, α is the road slope angle, ρ is the air density, $C_{w}$ is the drag coefficient, and $A_{window}$ is the vehicle’s windward area.

Various solutions have been offered to predict the front vehicle’s velocity for model predictive control (MPC), such as the Coordinated Leading Guidance (CLG) method [7] and the Nonlinear Auto Regressive with Exogenous Inputs (NARX) method [15]. These algorithms are constructive, but they have some limitations. Using these algorithms for systems with noise may lead to noise accumulation in multi-step predictions, resulting in inaccurate predictions.

The BPNN is combined with the forward vehicle speed prediction model proposed in the current study. As an example, we improve the NARX model suggested in [15]. The NARX and the BPNN are used for single-step and multi-step predictions, respectively. The BPNN aims to fit the possible increments for each step. The BPNN’s input is the front vehicle’s velocity from time $k-p_{p}$ to time k, defined as follows:

(7) $$h_{pre}≜\left[{\hat{V}}_{f,k-p_{h}|k-p_{h}},\cdots ,{\hat{V}}_{f,k-1|k-1},{\hat{V}}_{f,k|k}\right]$$

where $p_{h}$ represents the setting historical horizon.

**Figure 2:**
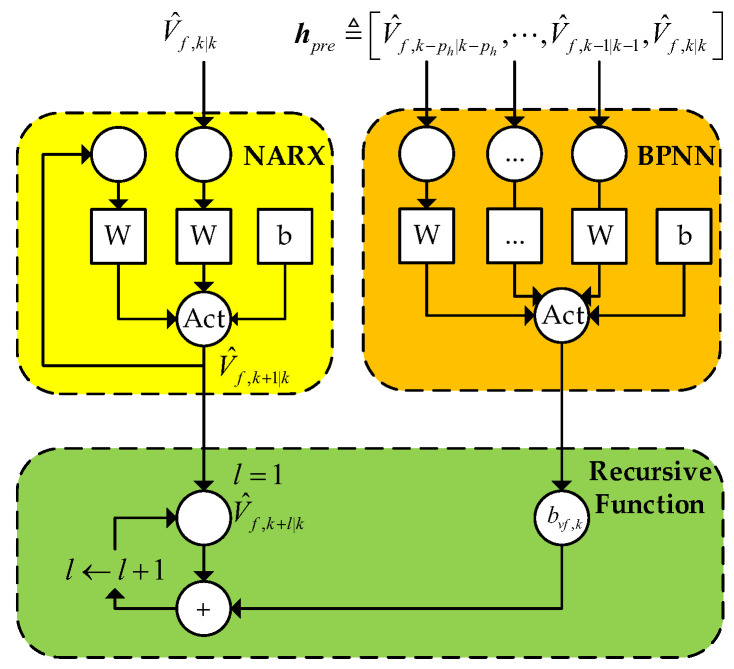
Structure diagram of the velocity of the front vehicle prediction model.

The structure of the model for front vehicle velocity prediction is shown in Figure 2, where “Act” represents the activation layer.

Using this approach, the l step at time k can be predicted as follows:

(8) $${\hat{V}}_{f,k+l|k}=\mathrm{NARX}\left({\hat{V}}_{f,k|k}\right)+\left(l-1\right)\mathrm{BPNN}\left(h_{pre}\right)$$

where $\mathrm{NARX}\left({\hat{V}}_{f,k|k}\right)$ and $\mathrm{BPNN}\left(h_{pre}\right)$ denote the predictions of the NARX and the BPNN, respectively.$\mathrm{NARX}\left({\hat{V}}_{f,k|k}\right)$ and $\mathrm{BPNN}\left(h_{pre}\right)$ are considered special functions. Equation (8) can be converted into a recursion formula as follows:

(9) $${\hat{V}}_{f,k+l|k}=\left\{\begin{array}{l} \mathrm{NARX}\left({\hat{V}}_{f,k|k}\right)\;\;\;\;\;\;\;\;\;\;\;\;\;\;\;\;\;\;\;\;\;l=1\\ {\hat{V}}_{f,k+l-1|k}+\mathrm{BPNN}\left(h_{pre}\right)\;\;\;\;\;l>1 \end{array}\right.$$

Furthermore, the relative distance between the front vehicle and the host vehicle can be calculated as follows:

(10) $$d_{k+l+1}=T_{s}\left({\hat{V}}_{f,k+l|k}-{\hat{V}}_{h,k+l}\right)$$

The approach used in the [7] algorithm of the CLG can also be applied here. These sequences of traffic light signals and the historical speed sequence of the previous vehicle’s velocity are combined and input into the BPNN. At the same time, the CLG is simplified to perform Bayesian network operations, executing only one step of prediction.

The above Equations (1)–(9) are ideal, but the actual situation is much more complicated. The primary sources of errors are:

Vehicle variation, which includes changes in the vehicle mass due to changes in the weight of the passengers and changes in the rolling resistance coefficient due to changing tires; environment variation, which includes changes in gravitational acceleration, wind resistance coefficient, and air density; and modeling errors, which include the effect of lateral direction control on velocity [8], the effect of state of charge (SOC) changes on battery voltage [22], and the effect of tire pressure on grip during acceleration and braking. The velocity of the front vehicle cannot be directly modeled, and the prediction of speed must have errors. Although considering more variables results in less process noise, accounting for too many factors will cause the matrix dimension to become too large.

Therefore, a balance between accuracy and complexity should be struck. The first two points affect the parameters in Equations (2) and (4)–(6). There are specific patterns of change over a short period of time. The last point causes random noise. These errors are described using $w_{k}$, thus $w_{k}$ is an additive noise with a non-zero mean that can be considered to obey a Gaussian process. Section 3.1 will demonstrate that the filter is statistically consistent between the Gaussian process assumption and the actual situation through a series of tests [23,24].

According to the series of derivations above, the EACC system process equation can be written as follows:

(11) $$x_{k+1}^{}=\left[\begin{array}{c} V_{f,k+1}\\ d_{k+1}\\ V_{h,k+1}\\ a_{h,k+1} \end{array}\right]=f\left(x_{k}^{},u_{k}\right)+w_{k}=\left[\begin{array}{c} \mathrm{NARX}{\left(V_{f,k}\right)}_{}\\ T_{s}V_{f,k}+d_{k}-T_{s}V_{h,k}\\ V_{h,k}+T_{s}a_{h,k}\\ \frac{\frac{T_{k}\left(u_{k}\right)i\eta_{}^{}}{r}-F_{rolling}-F_{air}\left(V_{h,k}\right)-F_{grade}}{m} \end{array}\right]+w_{k}$$

where $\mathrm{BPNN}\left(h_{pre}\right)$ in Equation (9) is not used in (11) but is used to make predictions in the controller, which will be described in detail in Section 3.2.

### 2.2. Vehicle Sensor Measurement Model

The goal of this section is to deduce the measurement equation by modeling sensors. Typically, most vehicles are equipped with a comprehensive set of sensors. The FMCW radar measures the relative velocity and distance between two vehicles, the wheel velocity sensor provides the host vehicle’s velocity, and the inertial measurement unit supplies the host vehicle’s acceleration.

Assuming that the measurement noise covariances $v_{k}$ for these sensors are Gaussian white noise with known values, the values of the measurement noise covariances can be obtained from sensor manuals or prior studies. The initial measurement noise covariance diagonal matrix $Q_{v0}$ is given by [16,25,26]:

(12) $$Q_{v0}=\mathrm{diag}\left(0.055,0.28,1,0.005\right)$$

However, this assumption may not hold in actual situations, where the measurement noise covariances may vary over time and have a non-zero mean. For example, severe tire slippage on wet roads can increase the wheel velocity sensor’s error [25]. Likewise, rain and fog may cause increased noise, reducing the radar’s penetration effect [18,27,28]. This situation is described in Section 3.1.

The EACC system measurement equation is written as follows:

(13) $$z_{k}=H_{k}x_{k}+v_{k}=\left(\begin{array}{cccc} 1 & 0 & -1 & 0\\ 0 & 1 & 0 & 0\\ 0 & 0 & \frac{30}{\pi r} & 0\\ 0 & 0 & 0 & 1 \end{array}\right)x_{k}+v_{k}$$

where 30/πr is the conversion of velocity (unit: m/s) to wheel velocity (unit: rpm).

### 2.3. Energy Model

In this section, the equations for energy consumption and recovery are derived. The efficiency of the motor at the working points is given by:

(14) $$P_{m}^{}=\left\{\begin{array}{l} \frac{T_{m}\left(u_{k}\right)n_{k}}{9549\eta_{m}^{}\left(T_{m}\left(u_{k}\right),n_{k}\right)}\;\;\;\;\;\;\;\;\;\;\;\;\;\;\;\;\;\;\;\;\;T_{m}\left(u_{k}\right)\geq 0\\ \frac{T_{m}\left(u_{k}\right)n_{m,k}}{9549}\eta_{m}^{}\left(T_{m}\left(u_{k}\right),n_{k}\right)\;\;\;T_{m}\left(u_{k}\right)\leq 0 \end{array}\right.$$

where $n_{k}$ is the motor’s rotation speed, $\eta_{m}^{}\left(T_{m}\left(u_{k}\right),n_{k}\right)$ is the electrical energy conversion efficiency, a quantity related to the motor speed and torque, and 9549 is the coefficient of unit conversion. Equation (14) is adapted from [15].

The KERS can recover some of the vehicle’s kinetic energy during braking and convert it into electrical energy. The braking process is divided into two stages based on braking strength [1,29]:pure electric braking and electro-hydraulic hybrid braking. The hybrid braking stage uses a combination of motor braking and hydraulic braking. The first stage saves more energy than the second stage because hydraulic braking converts part of the kinetic energy into thermal energy, resulting in energy waste.

In this paper, when $u_{k}$ is positive, the motor performs energy output; when it is negative, it signifies that the KERS performs energy recovery. Furthermore, $u_{k}=1$ and $u_{k}=-1$ represent the maximum throttle opening and the critical point between the two stages of braking, respectively. In this paper, the output torque and the input control quantity are considered linearly related, so the torque output function $T_{k}\left(u_{k}\right)$ and the control input $u_{k}$ exhibit the following relationship:

(15) $$T_{m}\left(u_{k}\right)=\left\{\begin{array}{l} u_{k}T_{m}^{\left(+\right)}u_{k}>0\\ u_{k}T_{m}^{\left(-\right)}u_{k}<0 \end{array}\right.$$

where $T_{m}^{\left(+\right)}$ and $T_{m}^{\left(-\right)}$ are the maximum torque for energy output and energy recovery input, respectively. Since only one set of gears exists for electric vehicles, the relationship between motor speed $n_{k}$ and vehicle velocity $V_{f,k}$ is as follows:

(16) $$V_{h,k}=\frac{n_{k}\pi r}{30i}$$

Substituting (15) and (16) into (14) yields:

(17) $$P_{m}^{}\left(u_{k},V_{h,k}\right)=\left\{\begin{array}{l} \frac{10u_{k}T_{m}^{\left(+\right)}iV_{h,k}}{3183\pi r\eta_{m}^{}\left(u_{k},V_{h,k}\right)}\;\;\;\;\;\;\;\;\;\;\;\;\;\;\;\;\;\;\;\;\;u_{k}\geq 0\\ \frac{10u_{k}T_{m}^{\left(-\right)}iV_{h,k}}{3183\pi r}\eta_{m}^{}\left(u_{k},V_{h,k}\right)\;\;\;\;\;\;u_{k}\leq 0 \end{array}\right.$$

**Figure 3:**
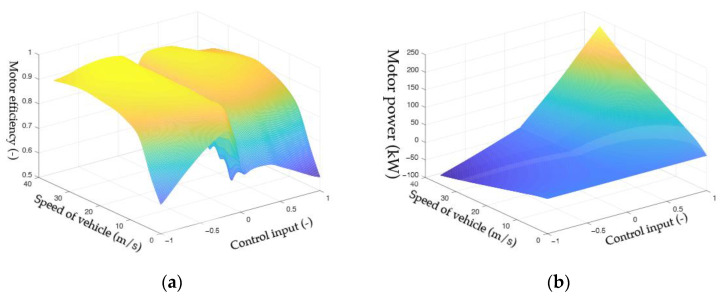
(**a**) Interpolation fitting surface for the relationship between motor efficiency and control input and motor speed; (**b**) The surface of the relationship between motor power and control input and motor speed.

Since the efficiency function $\eta_{m}^{}\left(u_{k},V_{h,k}\right)$ is nonlinear, the equation is fitted using Thin-plate spline interpolation, and the final result is shown in Figure 3a. The $P_{m}^{}\left(u_{k},V_{h,k}\right)$ obtained by Equation (17) is shown in Figure 3b.

Similar fitting methods can be applied to hybrid vehicles, with special consideration required for the product of the gearbox and main gearbox ratios.

## 3. Filter and Controller Algorithm

### 3.1. Filter Algorithm

Equations (11) and (13) present challenges for conventional nonlinear filtering methods, such as the extended Kalman filter (EKF) and the cubature Kalman filter (CKF) [30], as they assume that the noise is zero-mean Gaussian white noise with fixed covariance. Additionally, linear adaptive filtering methods, including the Sage-Husa adaptive Kalman filter (SHAKF) [31,32,33], cannot handle nonlinear functions. To address these limitations, we propose the SHACKF, a combination of the CKF and the SHAKF. The CKF approximates nonlinear functions using cubature points. The SHAKF algorithm parameters are updated as follows:

(18) $${\hat{q}}_{w,k}=\left(1-\delta_{k-1}\right){\hat{q}}_{w,k-1}+\delta_{k-1}\left({\hat{x}}_{k|k}-A_{k}{\hat{x}}_{k-1|k-1}\right)$$

(19) $${\hat{Q}}_{w,k}=\left(1-\delta_{k-1}\right){\hat{Q}}_{w,k-1}+\delta_{k-1}\left(K_{k}\epsilon_{k}\epsilon_{k}^{T}K_{k}^{T}+P_{k|k}-A_{k}P_{k|k}A_{k}\right)$$

(20) $${\hat{q}}_{v,k}=\left(1-\delta_{k-1}\right){\hat{q}}_{v,k-1}+\delta_{k-1}\left({\hat{z}}_{k|k}-H_{k}{\hat{x}}_{k|k-1}\right)$$

(21) $${\hat{Q}}_{v,k}=\left(1-\delta_{k-1}\right){\hat{Q}}_{v,k-1}+\delta_{k-1}\left(\epsilon_{k}\epsilon_{k}^{T}-H_{k}P_{k|k-1}H_{k}^{T}\right)$$

where ${\hat{q}}_{w,k}$ and ${\hat{Q}}_{w,k}$ denote the estimated process noise mean and covariances, respectively; ${\hat{q}}_{v,k}$ and ${\hat{Q}}_{v,k}$ denote the estimated measurement noise mean and covariances, respectively; $\epsilon_{k}=z_{k}^{}-{\hat{z}}_{k|k-1}^{}-q_{v,k}$ is the residual vector; $\delta_{k-1}=\left(1-b\right)/\left(1-b^{k+1}\right)$ and b represent the amnestic factor and the forgetting factor, respectively; $A_{k}$ and $H_{k}$ are the state transition matrix and measurement matrix, respectively; $K_{k}$ and $P_{k|k-1}$ are the Kalman gain and predicted error covariance matrix, respectively; and ${\hat{z}}_{k|k}$, ${\hat{x}}_{k|k-1}$, and ${\hat{x}}_{k|k}$ are the predicted measurement, predicted state, and posterior state, respectively.

To address this challenge, the SHACKF integrates the CKF and the SHAKF into a unified framework. The update process for the SHACKF is given in the following steps:

(22) $${\hat{q}}_{w,k}=\left(1-\delta_{k-1}\right){\hat{q}}_{w,k-1}+\delta_{k-1}\left({\hat{x}}_{k|k}-\frac{1}{2n}\sum_{\mu =1}^{2n}Z_{\mu ,\;k|k-1}^{}\right)$$

(23) $${\hat{Q}}_{w,k}=\left(1-\delta_{k-1}\right){\hat{Q}}_{w,k-1}+\delta_{k-1}\left(K_{k}\epsilon_{k}\epsilon_{k}^{T}K_{k}^{T}+P_{k|k}-\left(\frac{1}{2n}\sum_{\mu =1}^{2n}X_{\mu ,\;k|k-1}^{}{\left(X_{\mu ,\;k|k-1}^{}\right)}_{}^{Τ}-\;{\hat{x}}_{k|k-1}^{}{\left({\hat{x}}_{k|k-1}^{}\right)}_{}^{Τ}\right)\right)$$

(24) $${\hat{q}}_{v,k}=\left(1-\delta_{k-1}\right)q_{v,k-1}+\delta_{k-1}\left({\hat{z}}_{k|k}-\frac{1}{2n}\sum_{\mu =1}^{2n}Z_{\mu ,\;k|k-1}^{}\right)$$

(25) $${\hat{Q}}_{v,k}=\left(1-\delta_{k-1}\right)Q_{v,k-1}+\delta_{k-1}\left(\epsilon_{k}\epsilon_{k}^{T}-\left(\frac{1}{2n}\sum_{\mu =1}^{2n}Z_{\mu ,\;k|k-1}^{}{\left(Z_{\mu ,\;k|k-1}^{}\right)}_{}^{Τ}-\;{\hat{z}}_{k|k-1}^{}{\left({\hat{z}}_{k|k-1}^{}\right)}_{}^{Τ}\right)\right)$$

where $Z_{\mu ,\;k|k-1}^{}$ and $X_{\mu ,\;k|k-1}^{}$ indicate propagated cubature point sand cubature points, respectively [30].

To enhance the stability of the SHACKF, the innovation sequence $\epsilon_{k}$ and its theoretical statistical features are used to make judgments. If the filtering process is abnormal, the noise parameters are reinitialized [31]. The judgment is shown below:

(26) $$\epsilon_{k}\epsilon_{k}^{T}>\gamma tr\left(E\left[\epsilon_{k}\epsilon_{k}^{T}\right]\right)=\gamma tr\left(P_{zz,k|k-1}\right)$$

where γ is a coefficient, which is taken as 1 in this paper.

**Figure 4:**
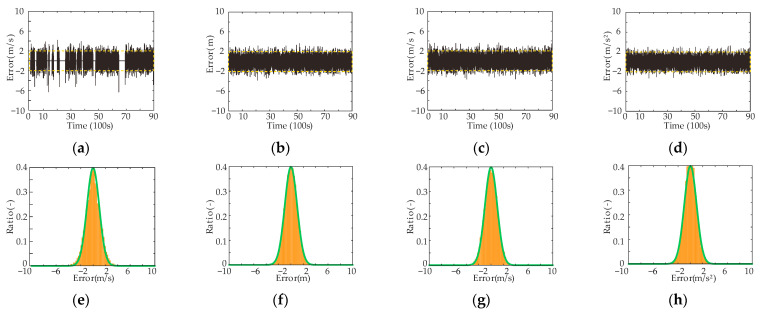
(**a**–**d**) The innovation sequences after normalization of the state of the system; (**e**–**h**) The distribution histograms.

As mentioned in Section 2.1, the error of the EACC system is assumed to obey a Gaussian process. The results are shown in Figure 4. Figure 4a–d show the autocorrelation of the normalized innovation sequence in the simulation [23]. The yellow dashed line represents the 95% confidence interval. All four components fall within the confidence interval of approximately 95%, which indicates that they are uncorrelated. Figure 4e–h represent the distribution histograms of the innovation sequence after the normalization of the four state components of the EACC system. The green lines represent the standard normal distribution. It can be seen that the four components are almost identical to the normal distribution. Therefore, there is sufficient reason to believe that the process noise obeys a Gaussian process.

However, there is an exception to the experiment. When the front vehicle stops, the vehicle velocity prediction at the next moment is also zero, which is almost correct and meaningless. This situation is reflected in the noiseless region in Figure 4a. Therefore, this situation is not considered in this paper.

### 3.2. MPC Controller Algorithm

**Figure 5:**
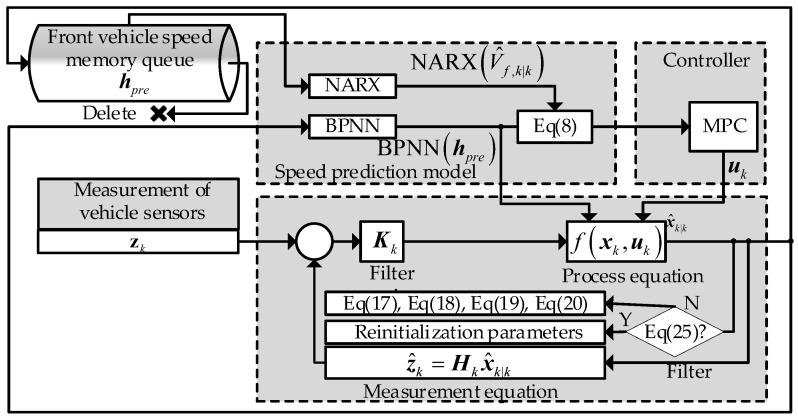
Schematic diagram of the algorithm proposed in this article.

In this section, the design of the control system is presented based on the previous discussion. The system framework is shown in Figure 5,and it consists of three main components: the front vehicle velocity prediction model, the filter, and the controller.

The front vehicle velocity prediction model, described in Section 2.1, uses a queue to store historical data and follows the FIFO (first-in, first-out) principle. When a new value arrives, it deletes the earliest value in the queue. This queue is initialized to zero.

The filter is described in Section 3.1. To begin, the system state is estimated by the CKF algorithm. Then, it determines whether the noise parameters need to be reinitialized according to Equation (26). If not, it updates them according to Equations (22)–(25). The values obtained after filtering are provided to the controller and the prediction model.

The controller uses an MPC algorithm and is designed based on the model in Section 2. It solves the optimal control input according to the current state estimation and the desired state. Then, the control input is applied to the system to achieve EACC. The slack variables are introduced into the MPC to reduce non-solutions, and the NMPC toolbox provided by MATLAB has been used to solve the nonlinear model predictive control problem. The loss function and constraints of the MPC problem are given by:

(27) $$\begin{array}{c} \min\\ {}^{\left|u_{k+l}\right|} \end{array}\varpi_{1}\sum_{l=1}^{p_{p}}T_{s}P_{m}^{}\left(u_{k+l},{\hat{V}}_{h,k+l|k}\right)+\varpi_{2}\sum_{l=1}^{p_{p}}\left|{\hat{V}}_{h,k+l|k}-{\hat{V}}_{f,k+l|k}\right|+\varpi_{3}\sum_{l=1}^{p_{p}}\left|{\hat{a}}_{k+l|k}\right|+\varpi_{4}\sum_{l=1}^{p_{p}}\left(\left|\varepsilon_{d}\right|+\left|\varepsilon_{V}\right|\right)$$

(27a) $${\hat{x}}_{k+l+1|k}^{}=f\left({\hat{x}}_{k+l|k}^{},u_{k+l}\right)$$

(27b) $$\underline{u}\leq u_{k+l}\leq \bar{u}$$

(27c) $$\underline{d}+\varepsilon_{d}<{\hat{d}}_{k+l|k}\leq \bar{d}-\varepsilon_{d}$$

(27d) $$0\leq {\hat{V}}_{h,k+l|k}\leq {\bar{V}}_{h}+\varepsilon_{V}$$

(27e) $$0\leq \varepsilon_{d}\leq {\bar{\varepsilon }}_{d}$$

(27f) $$0\leq \varepsilon_{V}\leq {\bar{\varepsilon }}_{V}$$

where $p_{{}^{p}}^{}$ is the MPC prediction horizon; ${\hat{V}}_{h,k+l|k}$ denotes the prediction of the speed of the preceding vehicle for k+l time under the premise of k time, and the same for the other similar subscripts; $\varepsilon_{d}$ and $\varepsilon_{V}$ represent the slack variables of relative distance and the host vehicle’s velocity, respectively; $\bar{d}$ and $\underline{d}$ represent the upper and lower bounds of the relative distance, respectively; $\bar{u}$ and $\underline{u}$ represent the upper and lower bounds of the control input, respectively; and ${\bar{V}}_{h}$ represents the upper bounds of the host vehicle’s velocity.

The four terms in Equation (27) consider energy consumption, the distance difference between the two vehicles, the velocity difference between the two vehicles, the host vehicle’s acceleration magnitude, and the slack variable magnitude. Equation (27a) is the process equation. The control input range is restricted by Equation (27b). The relative distance is restricted and maintained between cars by Equation (27c). The maximum vehicle velocity is limited by Equation (27d). The slack variable range is limited by Equations (27e) and (27f).

Although slack variables are introduced, there may be situations where no solution can be found. In such cases, the algorithm first tries to find a suboptimal solution. If that fails, the algorithm attempts to relax the energy, distance, or velocity constraint conditions. If that still fails, the algorithm reinitializes the MPC. This is because there may be an inconsistency between the control input and Equation (11) when the front vehicle completely stops. The reinitialization clears the previous input data and solves the problem again.

In summary, the proposed MPC controller algorithm addresses the challenge of EACC under noise uncertainty, and it effectively integrates the front vehicle velocity prediction model and the filter to achieve tracking control. Additionally, the algorithm can also be combined with studies [7,15].

## 4. Performance Analysis

The performance of the algorithm is analyzed in detail in this section using CarSim and Simulink. To solve the MPC problem, the NLMPC toolbox is utilized, and all simulations are conducted on a personal computer with an i5-8600 processor.

The host vehicle simulation model is based on the Tesla Model 3 Rear-Wheel Drive series with a single rear-mounted IPM-SynRM electric motor. Table 1 displays the simulation parameters for the host vehicle and environment. The “Model Value” column represents values used in the calculation, whereas the “Simulation Value” column denotes the values configured in the software during the simulation. This approach is reasonable since the electronic control unit cannot accurately measure relevant environmental parameters in real time. Table 2 shows the calculation parameters for the MPC.

To predict the front vehicle’s velocity, two types of neural networks, the BPNN and the NARX, are trained. The training data set is collected by Beamng.tech [34], a free simulation software for the academic community. The data set consisted of seventeen scenarios with various vehicles, weather, congestion, and road conditions, each with a driving time ranging from 75 s to 5 min. Beamng.tech has been used in simulations in previous studies [34,35].

In order to conduct a comprehensive evaluation of the algorithm’s performance, comparative experiments are performed between the algorithms proposed in [7,15] and the proposed algorithm under the same conditions. The algorithm is comprehensively tested for energy consumption, tracking, velocity following, and filtering using two types of cycles: the NEDC and the CLTC.

The NEDC is a driving cycle with four urban segments and one suburban segment, lasting 1180 s and covering a distance of 11.022 km. The average velocity is 33.6 km/h. The CLTC [36], short for China Light Vehicle Test Cycle, is composed of urban, suburban, and expressway segments with a total duration of 1800 s and a total distance of 14.48 km, with more random velocity variation than the NEDC.

### 4.1. Analysis of Energy Consumption

**Figure 6:**
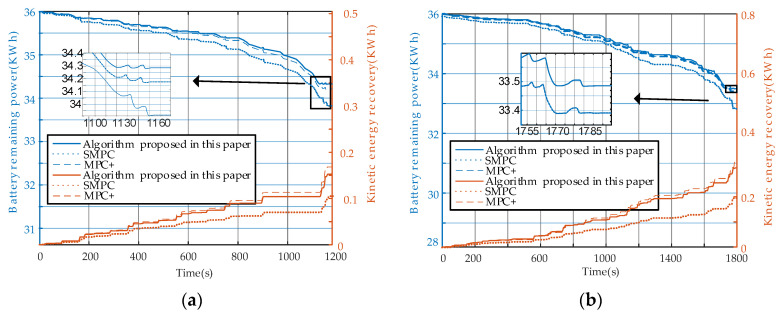
(**a**) Electricity consumption comparison under NEDC conditions; (**b**) Electricity consumption comparison under CLTC conditions.

In this section, the energy consumption of the host vehicle is analyzed. The stochastic model predictive control (SMPC) method proposed by Moser, D. et al. [7] and the MPC+ method proposed by Zhou, H. et al. [15] are compared with the proposed algorithm under the same conditions. The host vehicle has a total battery capacity of 60 kWh, with 36 kWh remaining at the initial moment. Figure 6a,b show the energy consumption and recovery under the NEDC and CLTC driving cycles, respectively.

The energy consumption gap between the proposed algorithm and the algorithms in [7,15] is mainly due to the inaccurate estimation. It is worth mentioning that the KERS in this algorithm recovers less energy than that of the algorithm proposed in [15], as the inaccurate prediction of the front vehicle’s velocity leads to frequent deceleration. Since the efficiency of the vehicle is less than 100%, accelerating and then decelerating to the same speed leads to a waste of energy. The study in [7] can be applied in the context of CACC or when communication is absent. However, this paper does not take into account the kinetic energy recovery system, which results in significantly lower energy recovery compared to the algorithms proposed in this paper and [15]. Additionally, higher energy consumption is observed due to the inaccuracies in the estimation.

The PID algorithm is used to simulate the vehicle’s driving under NEDC and CLTC conditions. The simulation results are considered the standard energy consumption, and the results are presented in Table 3.

The CLTC driving cycle has more complex environmental conditions than the NEDC, necessitating a more accurate front vehicle velocity prediction model. Inaccurate predictions can degrade overall performance. Noise filtering is crucial for the EACC system, as an accurate prediction of the dynamic system is a prerequisite for stable and efficient MPC operation [37]. All EACC algorithms consume more energy than the standard under the CLTC driving cycle due to the more complex and error-prone velocity prediction in the tracking case. However, by filtering and improving the EACC system, more energy can be saved by the algorithm proposed in this paper. Compared to the algorithm proposed in [15], the algorithm proposed in this paper can achieve an energy saving of 0.095 kWh per single CLTC. With a 60 kWh battery, the algorithm in [15] can run 347 km, the algorithm in [7] can run 289 km, and the algorithm in this paper can run 334 km. Energy can therefore be saved by 0.006 kWh/km and 0.03 kWh/km relative to the algorithms proposed in [7,15], respectively.

### 4.2. Analysis of Tracking Performance

**Figure 7:**
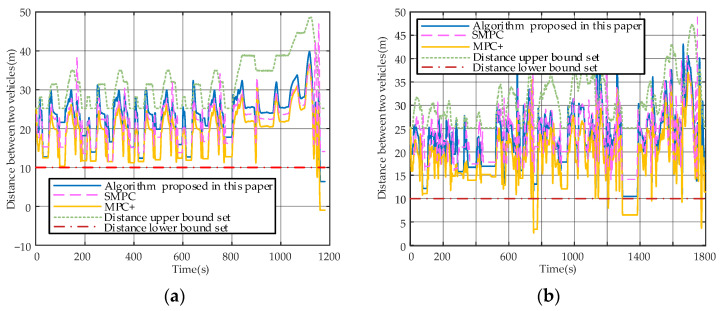
(**a**) Inter-vehicle distance tracking curve under NEDC conditions; (**b**) Inter-vehicle distance tracking curve under CLTC conditions.

**Figure 8:**
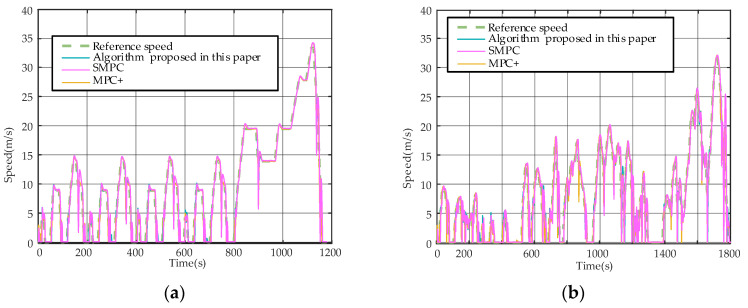
(**a**) Speed-tracking curve under NEDC conditions; (**b**) Speed-tracking curve under CLTC conditions.

The tracking performance of the vehicle is analyzed in this section. Figure 7a,b illustrate the inter-vehicle distance tracking under the NEDC and CLTC driving cycles, respectively. The green line represents the upper distance boundary, while the red line represents the lower distance boundary. Similarly, Figure 8a,b show the velocity tracking curves.

Constraint violations are observed in Figure 7a,b, particularly during the leading vehicle’s acceleration and deceleration phases, resulting from the inaccurate prediction of the front vehicle’s velocity. A method is proposed for a condition that failed to solve the MPC equation in this paper, allowing the vehicle to track or stop within acceptable limits even in violation of constraints. In contrast, the algorithms proposed in [7,15] do not consider this problem and noise, which led to over-near distance and even rear-end collisions at the last moment (1160 s) during the NEDC simulation.

Figure 6a,b show that all three algorithms predicted that the velocity continued to increase after almost all end-of-ascent time moments (e.g., 143 s, 338 s, and 533 s under NEDC conditions; 723 s, 998 s, and 1057sunderCLTC conditions). After a change occurred, they rapidly decelerated to follow the front vehicle’s velocity. Compared with [15], a variable distance limit is introduced, while a fixed one is used in [15]. The relative distances are similar at relatively low velocities, but the developed algorithm has a farther distance than [15] at faster velocities. Most of the relative distance differences between the two algorithms are within 5 m, which is acceptable. However, fixed limits may have led to prediction errors with noise, resulting in dangerous distances (e.g., 751 s, 1293 s, and 1774 s in the CLTC; 1160sin the NEDC, as mentioned earlier). The dangerous distance situations did not occur in [7], which can be attributed to the fact that the algorithm in [7] has no limit on the KERS and applies significant braking when approaching dangerous distances, leading to energy consumption.

By analyzing the tracking capabilities of all algorithms, it is evident that the proposed method minimizes the occurrence of accidents and enhances system reliability. However, this may introduce a computational burden. Each calculation time is recorded, with an average calculation time of 0.1141 s. This is still sufficient for current mainstream vehicle chips.

### 4.3. Analysis of Modeling and Filtering

In this section, the model developed in Section 2 and the SHACKF proposed in Section 3.1 will be analyzed.

**Figure 9:**
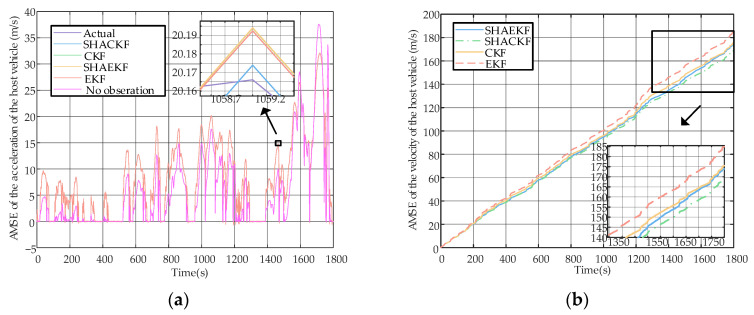
(**a**) Filtering and modeling algorithms for the host vehicle speed estimation curve; (**b**)AMSE of the host vehicle speed estimation curve.

Figure 7a shows the estimation of the velocity of the host vehicle by filtering and modeling algorithms. As can be seen from the Figure 9, several filtering algorithms are effective, as almost all are the same as the true value. The modeling algorithm does not use the observations to adjust the estimates, and this curve is always around the true value, which also proves that the model proposed in Section 2 is valid.

Figure 7b shows the accumulative mean square error (AMSE) of the SHAKF, SHACKF, CKF, and EKF, where the AMSE is as follows:

(28) $$\mathrm{AMSE}\left(k\right)=\sum_{t=0}^{k}{\left\|{\hat{x}}_{t|t}^{}-x_{t}\right\|}_{2}^{2}$$

In the figure, we can see that the SHACK algorithm has the best filtering performance, the CKF and the SHAKF are almost the same, and the EKF algorithm is the worst. It is understandable that the CKF uses the nonlinear property of the model, the SHAKF compensates for the error, and the SHACKF combines the advantages of these two algorithms.

## 5. Conclusions

In this paper, the design of anEACC system considering vehicle process and measurement noise is studied, focusing on how to minimize the impact of noise on the EACC system and save more energy. This study addresses non-zero mean noise in nonlinear vehicle systems by employing the SHACKF algorithm, which integrates the CKF and the SHAKF algorithms. To boost multi-step prediction accuracy, the vehicle speed prediction model is augmented with a BPNN. The issue of frequent acceleration and deceleration is tackled by considering vehicle kinetic energy recovery systems and interpolating motor efficiency. Furthermore, slack variables are employed to manage the infeasibility of MPC.

The simulation results provided valuable insights into the performance of our proposed algorithm. In terms of energy savings, the algorithm demonstrated significant improvements compared to previous methods, with numerical results showing an energy saving of 0.006–0.03 kWh/km. The proposed algorithm also outperformed previous algorithms in terms of stability in distance tracking, maintaining a consistent and appropriate distance from the leading vehicle. In velocity tracking, minor fluctuations were observed only during the final stages of acceleration and deceleration, while the tracking remained stable throughout other periods. It is worth mentioning that this algorithm is versatile and can be integrated with various current MPC-based EACC studies.

Future research may explore incorporating this algorithm into CACC studies by using information fusion algorithms to enhance traffic efficiency. Additionally, although the SHACKF algorithm is effective, it has the drawback of estimating covariance and mean inaccurately. To address this issue, more precise filters, such as robust adaptive filters and H∞ filters, could be employed to eliminate noise.

## Figures and Tables

**Table 1:** Vehicle and environmental parameters.

Parameter	Symbol	Model Value	Simulation Value	Unit
Maximum torque for energy output	$T_{m}^{+}$	340		Nm
Maximum torque for energy recovery input	$T_{m}^{-}$	135		Nm
Tire specification	-	235/55 R18	225/55 R18	-
Air resistance coefficient	$C_{w}$	0.23		-
Transmission ratio	i	7.2288		-
Vehicle and passenger mass	m	1440	1580	kg
Windward area	$A_{window}$	0.7035		m^2^
Rolling resistance coefficient	-	0.9	0.75	-

**Table 2:** The parameters of the MPC algorithm.

Parameter	Symbol	Value		Unit
Historical horizon	$p_{h}$	10		-
Prediction horizon	$p_{p}$	10		-
Relative distance, upper bound	$\bar{d}$	$25+0.7V_{f,k}$		m
Relative distance, lower bound	$\underline{d}$	10		m
Sampling time	$T_{s}$	0.5		s
Host vehicle’s velocity, upper bound	${\bar{V}}_{h}$	$V_{f,k}+5$		m/s
Upper bound on the relaxation variable of the distance	$\varepsilon_{d}$	0.5		m
Upper bound on the relaxation variable of the velocity	$\varepsilon_{V}$	0.3		m/s
Weights	$\varpi_{1}$	$\varpi_{2}$	$\varpi_{3}$	$\varpi_{4}$
	0.1	1	0.6	0.01

**Table 3:** Comparison of energy consumption and recovery.

Circulation	Energy Consumption in This Paper	Energy Consumption in [15]	Energy Consumption in [7]	Energy Recovery in This Paper	Energy Recovery in [15]	Energy Recovery in [7]	Standard
Unit	kWh	kWh	kWh	kWh	kWh	kWh	kWh
NEDC	1.6728	1.786	2.0923	0.1526	0.1682	0.1035	1.926
CLTC	2.505	2.6000	3.0070	0.2862	0.3087	0.1921	2.154

## Data Availability

Not applicable.
